# Pediatric Critical Care Medicine Training in India: Past, Present, and Future

**DOI:** 10.3389/fped.2018.00034

**Published:** 2018-02-26

**Authors:** Utpal Bhalala, Praveen Khilnani

**Affiliations:** ^1^Baylor College of Medicine at the Children’s Hospital of San Antonio, San Antonio, TX, United States; ^2^Rainbow Children’s Hospital, New Delhi, India

**Keywords:** pediatric critical care, training, fellowship, simulation, India, resource-limited settings

## Abstract

Pediatric critical care services in India have grown with leaps and bounds. There has been a growing need of physicians specially trained in pediatric critical care medicine (PCCM) in India. Physicians returning to India after their formal training in PCCM abroad have partly supported this growing need. Development of formal PCCM training programs in India has been a huge step toward supporting the growing clinical needs. This article focuses on advances in pediatric critical care training in India and its future directions.

## Introduction

Until 2002, formal fellowship training in pediatric critical care medicine (PCCM), accredited by a national governing body did not exist in India. Therefore, pediatricians interested in such training, explored options outside of India for formal PCCM fellowship. Over the subsequent 15 years, the scenario of PCCM changed dramatically in India. Quite a few physicians after completing a formal PCCM fellowship and gaining some experience working as senior house officers or faculty abroad returned to India to lead the PCCM programs in major metropolitan areas of India. Also, a very good number of hospitals established formal fellowship training in PCCM. This article focuses on formal, curriculum-based PCCM training as well as recent advances in simulation-based PCCM training in India.

## Growth of Pediatric Critical Care Specialty in India (Figure 1)

**Figure 1:**
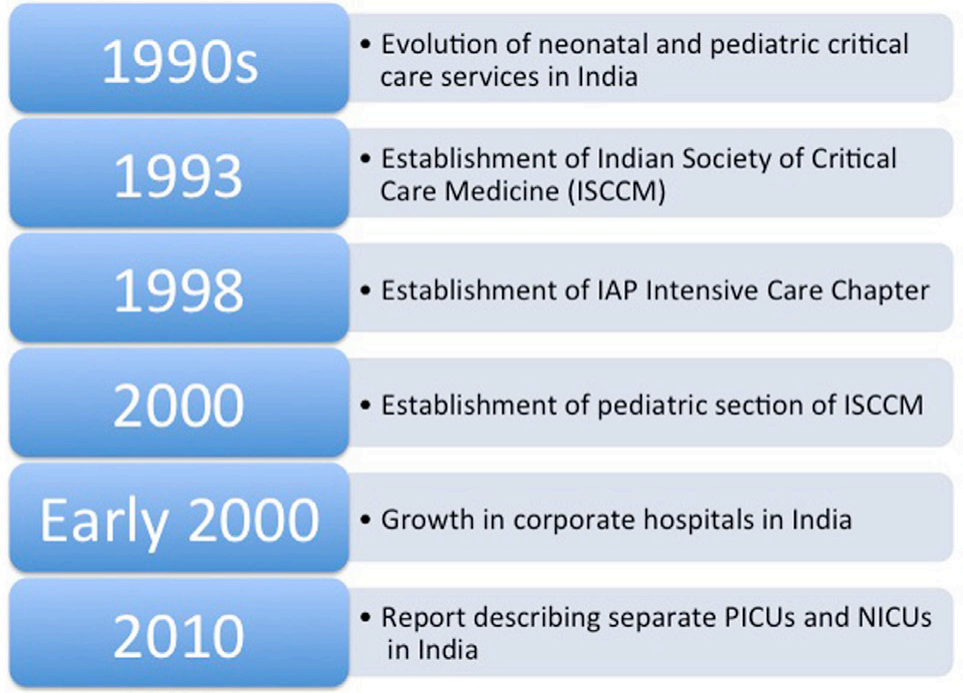
A flow diagram of growth of pediatric critical care medicine in India.

In 1997, Vidyasagar and co-authors summarized the evolution of neonatal and pediatric critical care in India (1) (Figure 1). The initiative to upgrade neonatal and pediatric critical care came primarily from major teaching hospitals, especially government sponsored institutions (1). Around the turn of the century, India witnessed a boom in the field of healthcare (2, 3). There was a significant growth in number of corporate hospitals capable of providing state-of-the-art services to patients in metropolitan areas of India (3). These corporate hospitals took the lead in establishing units based on Western standards of equipment and personnel, thereby moving the PCCM specialty in a favorable direction in India (1, 4). The establishment of these hospitals created newer opportunities and attracted well-trained and experienced physicians and surgeons to either return to India or move from government hospitals to corporate hospitals to establish specialty and subspecialty divisions. In 2010, Lodha and co-authors reported a few centers in government and private hospitals in India with separate pediatric intensive care units (PICUs) (5). Now there are multiple, small-to-medium size PICUs all over India, which are run by pediatric intensivists formally trained either abroad or within India. In fact, Dr. Khilnani under auspices of Indian Academy of Pediatrics (IAPs) published consensus guidelines for design and operation of PICUs in India (6). Needless to say, large proportions of children in rural and remote parts of India are still deprived of timely critical care services and succumb to the illness.

## Contributions of National Societies in Supporting the Pediatric Critical Care Training in India

Indian Society of Critical Care Medicine (ISCCM) was established in Mumbai, India in 1993. It is the largest non-profit association of Indian physicians, nurses, physiotherapists, and other allied health care professionals involved in the care of the critically ill. ISCCM, which started with a small group of intensivists, from Mumbai, has grown to membership of 7,440, comprising of 67 city branches all across the India with headquarter in Mumbai (7). The pediatric section of ISCCM was established in 2000. It has more than 200 members. Indian College of Critical Care Medicine was established by ISCCM to implement and carry out all the educational activities, including Indian fellowship in critical care medicine and Indian diploma in critical care medicine (7). The intensive care chapter of IAPs was established in 1998 (8). Since then, IAP Intensive Care Chapter has been successful in promoting the field of pediatric critical care. It has more than 500 members. The chapter has established guidelines for PICUs and education programs (8). ISCCM in coordination with the IAP intensive care chapter has developed PCCM fellowship with an established curriculum and training at the approved PICUs all over the country (9). The pediatric section of ISCCM and IAP has been active in beginning Diplomat in National Board certification in PCCM (7, 10). The national bodies have been active in organizing critical care conferences, workshops, and courses such as basic pediatric intensive care in India (8). This has not only improved networking opportunities connecting the potential PCCM fellows to the appropriate fellowship programs but also reinforced their clinical and academic training.

## PCCM Training in India

**Figure 2:**
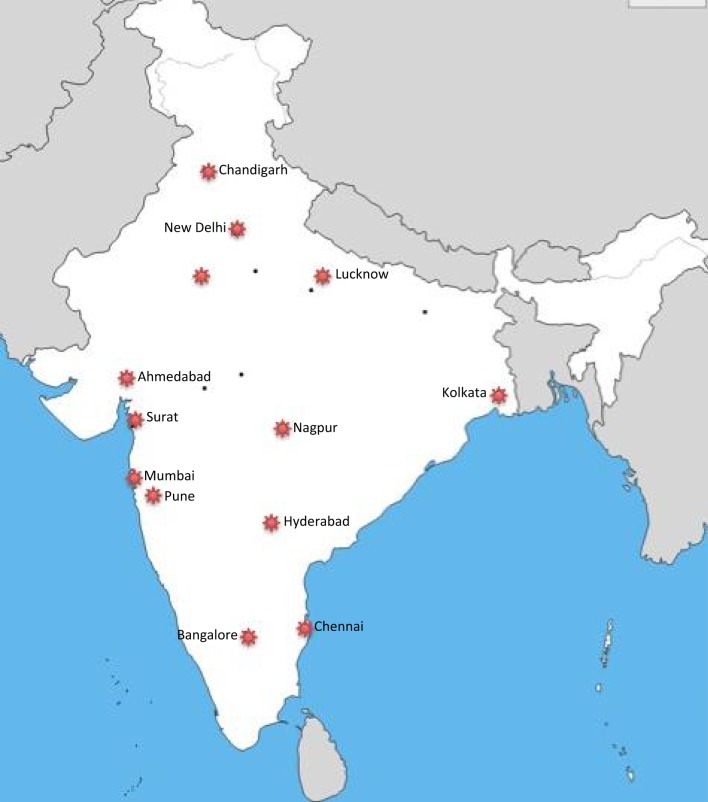
Map of major cities of India with pediatric critical care medicine fellowship training centers.

There are now 22 official PCCM fellowship programs listed on the college of pediatric critical care website and the number is growing every year (8) (Figure 2). There are different curriculum-based PCCM fellowships—a 1-year fellowship and a 2-year fellowship, each heavily clinically focused. There are only a handful of institutions in India, which have started a 3-year PCCM fellowship with curriculum similar to that of PCCM training in the West. The 3-year fellowship provides sufficient time for accomplishing a meaningful research project. The 3-year fellowship programs, which offer Doctor of Medicine degree upon successful completion of the fellowship, have been restricted to large, esteemed, government-run teaching institutions (10).

During fellowship, the fellows work closely with pediatric critical care consultants, pediatric cardiologists, surgeons, and other specialists. In India, traditional bedside teaching is still far more prevalent than classroom teaching. Apart from fundamental critical care training, the fellows also get exposed to counseling families, delivering difficult news, quality, and safety in PICU and end of life care. The fellows also get trained in designing a PICU, understanding a need for PICU in rural setting, creating, managing and maintaining a new PICU, and working on a budget for building a new PICU, implementing PICU protocols and a teaching program. Critical care ultrasound has been increasingly utilized in India (10–12). A significant number of PICUs in India have started using bedside ultrasound for assessment of critically ill children and monitoring of the therapies (13, 14). But the training of the PCCM fellows in India occurs largely based on the traditional “see one, do one and teach one” model. A handful of PCCM fellows attend critical care ultrasound workshop or course (15) but there is no formal curriculum or credentialing process for critical care ultrasound (16).

For PCCM fellows in India, majority of clinical learning, including procedural skills occur on the job. In the USA, a large number of PCCM fellowship training programs have adopted a formal fellow orientation process, which often includes hands-on airway skills in the operating room under supervision of a pediatric anesthesiologist and simulation-based training before they actually begin to work in the PICU. Similar to the West, majority of PCCM fellowship programs in India offer hands-on airway skills training in the operating room in the first year of fellowship. Recently, there is an increase in awareness and use of simulation-based teaching and training methodologies in pediatric emergency and critical care medicine.

## Simulation-Based PCCM Training in India

Over last 5 years, there is a significant growth in simulation-based PCCM training. Pediatric Simulation Training and Research Society (pediSTARS) has played vital role in introducing and spreading the simulation-based pediatric training in India (17). Back in 2011, the society introduced the first simulation workshop focused on pediatric emergencies as a pre-congress workshop prior to National Conference of Pediatric Critical Care (NCPCC) (17). But the simulation-based training in PCCM remained largely dormant in India for a couple of years beyond the initial attempt at introducing the concept of simulation. Our first regional pediatric critical care simulation workshop organized and conducted by us in Mumbai in 2013 (18) drew a significant interest and enthusiasm in simulation-based PCCM training in rest of the India. Ever since then, the IAP intensive care chapter adopted it as a standing pre-congress workshop for the NCPCC. Also, following this, pediSTARS launched the first national conference in pediatric simulation, SIMULUS along with the first pediatric simulation training of trainers’ workshop in 2014 in India (17). PediSTARS India has played a prominent role in organizing SUCCESS—Simulation Course for Critical care Emergencies for pediatric intensive care trainees (17). Moving forward, simulation-based PCCM training must focus on basic and advanced PCCM training in keeping with the challenges of critical care delivery unique to the developing world, such as limitation of resources (19), fulminant infections unique to the developing world (20) and transportation of critically ill children (21).

Simulation training of PICU fellows should also focus on training them on multidisciplinary care, prevention, and management of team conflicts, dealing with difficult situation, delivering bad news, dealing with hostile and uncooperative family.

## Future of PCCM Training in India

A rapid growth of PCCM training programs in India has paralleled and complemented the rapid growth of healthcare sector in India. Two national societies—ISCCM and IAPs have played pivotal role in growing and supporting PCCM fellowship programs all over India and in future they will continue to advance the field. In future, it is likely that a larger number of teaching institutions in India will adopt a 3-year fellowship curriculum with balanced clinical and academic PCCM training. Critical care ultrasound training, simulation-based training, training in advanced therapies, such as renal replacement and extracorporeal life support, training in quality and safety, and research methodologies are likely to become integral part of the PCCM fellowship in India in future. Western influence is likely to bring less hierarchy in healthcare. Therefore, a formal, 360° evaluation system within PCCM fellowship is likely to be introduced in India in future. Similar to what western countries witnessed in the last decade, PCCM subspecialty services in India, such as pediatric cardiac critical care and pediatric neurocritical care, are likely to grow and branch out from the general pediatric critical care. Separate pediatric cardiac intensive care units already exist in a good number of private and government hospitals since last few years. It is likely that special critical care training programs focused on pediatric cardiac critical care and pediatric neurocritical care will begin in India in the near future. In short, since the beginning of the new century, the PCCM training in India has grown with leaps and bounds and it is still growing. The contributions of national societies and quality of standards of PCCM fellowship programs maintained by national societies and governing bodies, such as ISCCM, IAP intensive care chapter, Medical Council of India, and National Board of Examinations, will define the future of pediatric critical care training in India.

## Author Contributions

Each author contributed equally to this manuscript.

## Conflict of Interest Statement

The authors declare that the research was conducted in the absence of any commercial or financial relationships that could be construed as a potential conflict of interest.
